# Diagnostic performance and image quality of an image-based denoising algorithm applied to radiation dose-reduced CT in diagnosing acute appendicitis

**DOI:** 10.1007/s00261-024-04246-3

**Published:** 2024-02-27

**Authors:** Hyeon Ui Choi, Jungheum Cho, Jinhee Hwang, Seungjae Lee, Won Chang, Ji Hoon Park, Kyoung Ho Lee

**Affiliations:** 1 Department of Radiology, Seoul National University Bundang Hospital, Seongnam-si, Gyeonggi-do Korea; 2 Department of Applied Bioengineering, Graduate School of Convergence Science and Technology, Seoul National University, Seoul, Korea; 3 Institute of Health and Environment, Seoul National University, Seoul, Korea; 4 Department of Radiology, Seoul National University College of Medicine, Seoul National University Bundang Hospital, Seongnam-si, Gyeonggi-do Korea; 5 Department of Medical Device Development, Seoul National University College of Medicine, Seoul, Korea

**Keywords:** Appendicitis, Tomography, X-Ray computed, Abdomen, Radiation dosage

## Abstract

**Purpose:**

To evaluate diagnostic performance and image quality of ultralow-dose CT (ULDCT) in diagnosing acute appendicitis with an image-based deep-learning denoising algorithm (IDLDA).

**Methods:**

This retrospective multicenter study included 180 patients (mean ± standard deviation, 29 ± 9 years; 91 female) who underwent contrast-enhanced 2-mSv CT for suspected appendicitis from February 2014 to August 2016. We simulated ULDCT from 2-mSv CT, reducing the dose by at least 50%. Then we applied an IDLDA on ULDCT to produce denoised ULDCT (D-ULDCT). Six radiologists with different experience levels (three board-certified radiologists and three residents) independently reviewed the ULDCT and D-ULDCT. They rated the likelihood of appendicitis and subjective image qualities (subjective image noise, diagnostic acceptability, and artificial sensation). One radiologist measured image noise, signal-to-noise ratio (SNR), and contrast-to-noise ratio (CNR). We used the receiver operating characteristic (ROC) analyses, Wilcoxon’s signed-rank tests, and paired t-tests.

**Results:**

The area under the ROC curves (AUC) for diagnosing appendicitis ranged 0.90–0.97 for ULDCT and 0.94–0.97 for D-ULDCT. The AUCs of two residents were significantly higher on D-ULDCT (AUC difference = 0.06 [95% confidence interval, 0.01–0.11; *p* = .022] and 0.05 [0.00–0.10; *p* = .046], respectively). D-ULDCT provided better subjective image noise and diagnostic acceptability to all six readers. However, the response of board-certified radiologists and residents differed in artificial sensation (all *p* ≤ .003). D-ULDCT showed significantly lower image noise, higher SNR, and higher CNR (all *p* < .001).

**Conclusion:**

An IDLDA can provide better ULDCT image quality and enhance diagnostic performance for less-experienced radiologists.

**Supplementary Information:**

The online version contains supplementary material available at 10.1007/s00261-024-04246-3.

## Introduction

With the technological advancements in CT imaging, the use of CT scans has rapidly increased worldwide [1, 2]. Concerns regarding the potential cancer risks implicated with radiation exposure from CT scans have also been raised, estimated to contribute to 1.5 to 2.0% of all cancers diagnosed in the United States [3]. Exposure to even a single abdominopelvic CT for diagnosing appendicitis scan may increase the risk of developing hematologic malignant neoplasms [4]. The imaging community has made several efforts to reduce the dose to as low as reasonably achievable; however, physicians and radiologists considered the trade-off of lower image quality unsatisfactory.

CT vendors have implemented various technologies to reduce radiation doses while maintaining image quality, such as automatic exposure control, noise reduction filters, and iterative reconstruction algorithms [5–7]. While iterative reconstruction played a critical role in preserving or enhancing image quality in low-dose CT examinations, it has limitations of being vendor-specific and not able to apply CT images generally. An alternative approach involves using vendor-agnostic image-based noise reduction strategies [8, 9]. Image-based methods are relatively inexpensive to install and can be useful in CT practices that employ multiple different scanner models [10], as a single server can service various CT scanners. Furthermore, recent advancements in deep-learning techniques have facilitated improvements in image-based denoising strategies, making them more versatile and applicable.

Compelling evidence suggests that CT can be used to diagnose appendicitis at a radiation level as low as 2 mSv, without negative ramifications [11–13]. In this study, we aimed to evaluate the diagnostic performance and image quality of 1-mSv CT (hereinafter, ultralow-dose CT [ULDCT]) with an image-based deep-learning denoising algorithm in patients with suspected appendicitis.

## Materials and methods

### Study design and participants

**Fig. 1:**
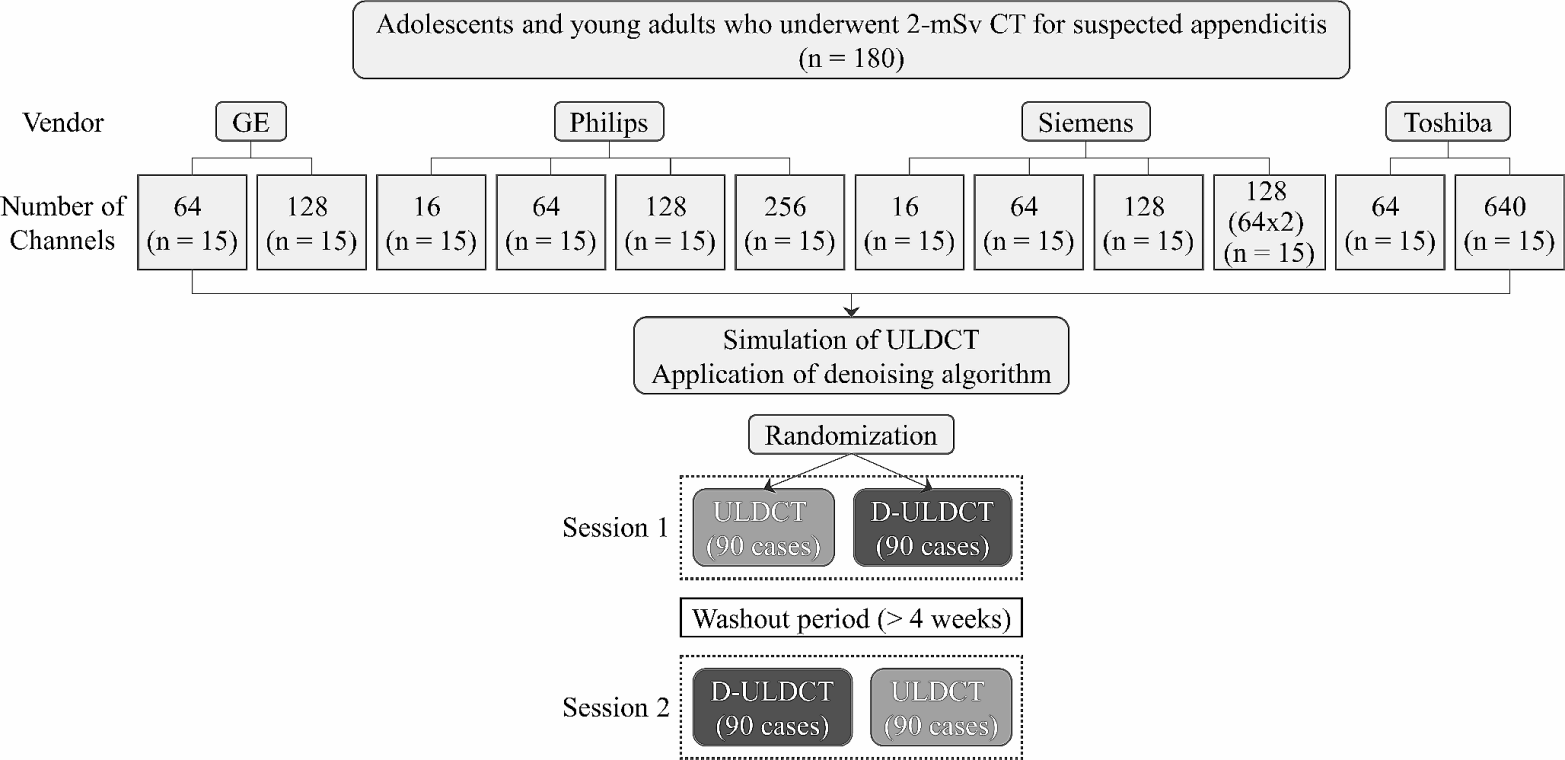
Flowchart of the study. *ULDCT* ultralow-dose CT, *D-ULDCT* denoised ultralow-dose CT

The institutional review board approved this retrospective study, and the requirement for informed consent was waived. We extracted clinical data and CT images of patients from a previous multicenter trial (Low-dOse CT for Appendicitis Trial, LOCAT; ClinicalTrials.gov number, NCT01925014) that demonstrated the non-inferiority of low-dose CT (with a target effective dose of 2 mSv) compared to standard-dose CT (with a target effective dose less than 8 mSv) in the diagnosis of appendicitis in adolescents and young adults [11]. The eligibility criteria for the trial were patients aged 15–44 years who were referred from the emergency departments for CT examination under the suspicion of appendicitis. The final diagnosis of appendicitis is based on the trial data, including surgical, pathologic, and follow-up results. In this study, we included 180 patients (15 to 44 years of age; 91 female) who underwent 2-mSv CT examinations from February 2014 to August 2016. We randomly selected 15 patients per each type of CT machine (Fig. 1). The characteristics of the study population are summarized in Table 1. We wrote this report in line with a reporting guideline (Standards for Reporting of Diagnostic Accuracy; STARD) [14].

**Table 1:** Baseline patient characteristics

Characteristics	
Age (years)	29 ± 9 (15–44)
Sex	
Female	91 (50.5%)
Male	89 (49.5%)
Body mass index (kg/m^2^)	22.5 ± 3.5 (14.7–35.5)
< 18.5 (underweight)	11 (6.1%)
18.5–24.9 (normal)	134 (74.4%)
≥ 25.0 (overweight or obesity)	35 (19.5%)
Diameter of the abdomen (cm)	
Anteroposterior	17.7 ± 3.0 (11.9–26.8)
Lateral	31.0 ± 3.9 (23.1–42.6)
Effective	23.4 ± 3.3 (16.8–33.5)
Diagnosis	
Acute appendicitis	58 (32.2%)
Perforated appendicitis	17 (9.4%)
Others	122 (67.8%)
Radiation dose	
Volume CT dose index (mGy)	2.6 ± 0.8 (1.3–7)
Dose-length product (mGy·cm)	139.3 ± 45.7 (71.0–351.0)
Size-specific dose estimate (mGy)	4.1 ± 1.0 (2.1–11.3)
CT machine (channels)	
16	30 (16.7%)
64	60 (33.3%)
128	45 (25.0%)
128 (64 × 2)	15 (8.3%)
256	15 (8.3%)
640	15 (8.3%)
CT manufacturer	
GE	30 (16.7%)
Philips	60 (33.3%)
Siemens	60 (33.3%)
Toshiba	30 (16.7%)

Data are mean ± standard deviation (range) or number (%)

### CT image acquisition

The patients underwent 2-mSv CT from machines with 16 to 640 channels from various manufacturers (64 and 128 channels from GE Healthcare; 16, 64, 128, and 256 from Philips; 16, 64, 128, and 128 (64 × 2) from Siemens; 64 and 640 from Toshiba). All patients underwent CT scans using intravenous contrast agents. The abdominopelvic CT images were obtained during the portal venous phase and reconstructed using filtered back projection with a slice thickness of 4 mm and a slice interval of 3 mm. The mean volumetric CT dose index was 2.6 ± 0.8 mGy, and the mean dose-length product was 139.3 ± 45.7 mGy·cm.

### Simulation of ultralow-dose CT and application of denoising algorithm

From the 2-mSv CT images, we simulated ULDCT images with an image-based reduced-dose CT simulation technique [15], reducing the dose by at least 50%. This technique is based on sinogram synthesis and image reconstruction using only CT images while not requiring raw sinogram data. Previous studies validated the dose reduction technique, which provided realistic low-dose images including the noise and textual appearance [16–18].

Then, we used an image-based deep-learning denoising algorithm (ClariCT.AI™, ClariPI) to generate denoised ULDCT (D-ULDCT) images [19]. This deep-learning algorithm is a vendor-neutral image reconstruction technique based on a modified U-net type convolutional neural network model [20]. The algorithm was trained using a dataset of over 1 million CT images, covering 2,100 combinations of scan and reconstruction conditions, including variations in kVp, mAs, automatic exposure control, slice thickness, contrast enhancement, and convolution kernels. The dataset encompassed 24 scanner models from four CT manufacturers (GE Healthcare, Siemens, Philips, and Canon). The algorithm’s performance has been validated in several studies [17, 21–24].

### Qualitative image analysis

Six radiologists with different experience levels (three board-certified abdominal radiologists with 6 to 7 years of clinical experience, one third-year, and two second-year residents) independently reviewed the ULDCT and D-ULDCT images. We randomly assigned 180 patients to two groups (90 patients in groups A and B, respectively). In the first session, each radiologist assessed the ULDCT images of Group A and the D-ULDCT images of Group B. In the second session, they evaluated the D-ULDCT images of Group A and the ULDCT images of Group B. To reduce recall bias, the first and second sessions were separated with a washout period of at least 4 weeks, and the order of the CT images was randomized and different for each session. All readers were informed that patients underwent CT examinations for suspected appendicitis but were blinded to other patient information, study date, radiation dose, and reconstruction algorithm.

Firstly, the radiologists were asked to rate appendiceal visualization and likelihood score for appendicitis using the standardized CT report form (Supplementary Table 1). Appendiceal visualization was rated using a 3-point Likert scale (grade 0, not identified; grade 1, unclearly or partially visualized; and grade 2, clearly and entirely visualized). If the CT image showed phlegm or abscess with clear continuity with the remaining appendiceal base, grade 2 was assigned. The likelihood score for appendicitis was rated on a 5-point Likert scale. The primary diagnostic criterion was appendiceal enlargement (larger than 6 mm in diameter) with mural thickening and periappendiceal fat stranding. Secondary diagnostic criteria were abnormal mural enhancement, appendicolith, phlegmon, and abscess. For diagnostic sensitivity and specificity calculation, the likelihood score for appendicitis ≥ 3 was considered positive for the diagnosis [25].

Secondly, the radiologists independently rated the image quality of ULDCT and D-ULDCT by using a 5-point Likert scale (Supplementary Table 2). The following attributes were evaluated: subjective image noise (defined as the degree of mottling or graininess in the images), diagnostic acceptability (defined as the reader’s confidence in making a reasonable diagnosis from the image), and artificial sensation (defined as the degree of plastic-looking, smooth, paint-brushed, or unnatural texture).

### Quantitative image analysis

A quantitative analysis of the image quality was conducted based on image noise, signal-to-noise ratio (SNR), and contrast-to-noise ratio (CNR) [26]. A board-certified radiologist with 7 years of abdominal imaging experience, who was independent of the six readers, measured the mean Hounsfield unit and standard deviation (SD) of the hepatic parenchyma, paraspinal muscle, abdominal aorta, and subcutaneous fat on ULDCT and D-ULDCT images using oval-shaped regions of interest (ROIs; 50 to 200 mm^2^ in size). Hounsfield unit of the hepatic parenchyma was obtained by averaging values of the four liver sections (left lateral, left medial, right anterior, and right posterior). The ROIs were placed in homogeneous areas at the level of the umbilical portion of the left portal vein, avoiding structures such as large vessels, intramuscular fat, or calcified vessel walls. The ROIs were positioned at the same location and size on ULDCT and D-ULDCT images from the same patient.

We calculated the image noise as the mean SD of the ROIs. SNR and CNR for each target region were determined using the following equations [26]:

SNR_i_ = ROI_i_ / SD_i_.

CNR_i_ = (ROI_i_ - ROI_fat_) / SD_fat_.

where ROI_i_ is the mean attenuation of the region, ROI_fat_ is the mean attenuation of the subcutaneous fat, SD_i_ is the image noise of the region, and SD_fat_ is the image noise of the subcutaneous fat.

***Reference standard***.

In previous trial, independent outcome assessors, who were two emergency department physicians and five radiologists, adjudicated the final diagnosis of appendicitis based on the trial data, including surgical findings, pathologic findings, and follow-up results [27]. All final diagnoses of appendicitis included in this study were established based on surgical and pathologic findings [11]. Histopathologic diagnosis of appendicitis was defined as neutrophil infiltration in the appendiceal wall [28].

### Statistical analysis

We used the Wilcoxon signed-rank test to compare appendiceal visualization from each reader between ULDCT and D-ULDCT. We used the receiver operating characteristic (ROC) analysis to assess the diagnostic accuracy of readers in diagnosing appendicitis. We compared the area under the receiver-operating characteristic curve (AUC) of each reader between ULDCT and D-ULDCT using DeLong’s test for two correlated ROC curves. Additionally, we calculated diagnostic sensitivity and specificity in the ULDCT and D-ULDCT. For interobserver agreement, we calculated the quadratic weighted Kappa coefficient for board-certified radiologists and residents regarding ULDCT and D-ULDCT. We used z tests to compare the interobserver agreement between ULDCT and D-ULDCT.

We used the Wilcoxon signed-rank test to compare the subjective qualitative scores between ULDCT and D-ULDCT in each reader and the paired t-test to compare quantitative parameters (image noise, SNR, and CNR) between ULDCT and D-ULDCT.

Statistical analyses were performed using R software version 3.6.3 (www.R-project.org, R Foundation for Statistical Computing). A two-sided *p*-value of < 0.05 was considered statistical significance.

## Results

### Qualitative image analysis

**Fig. 2:**
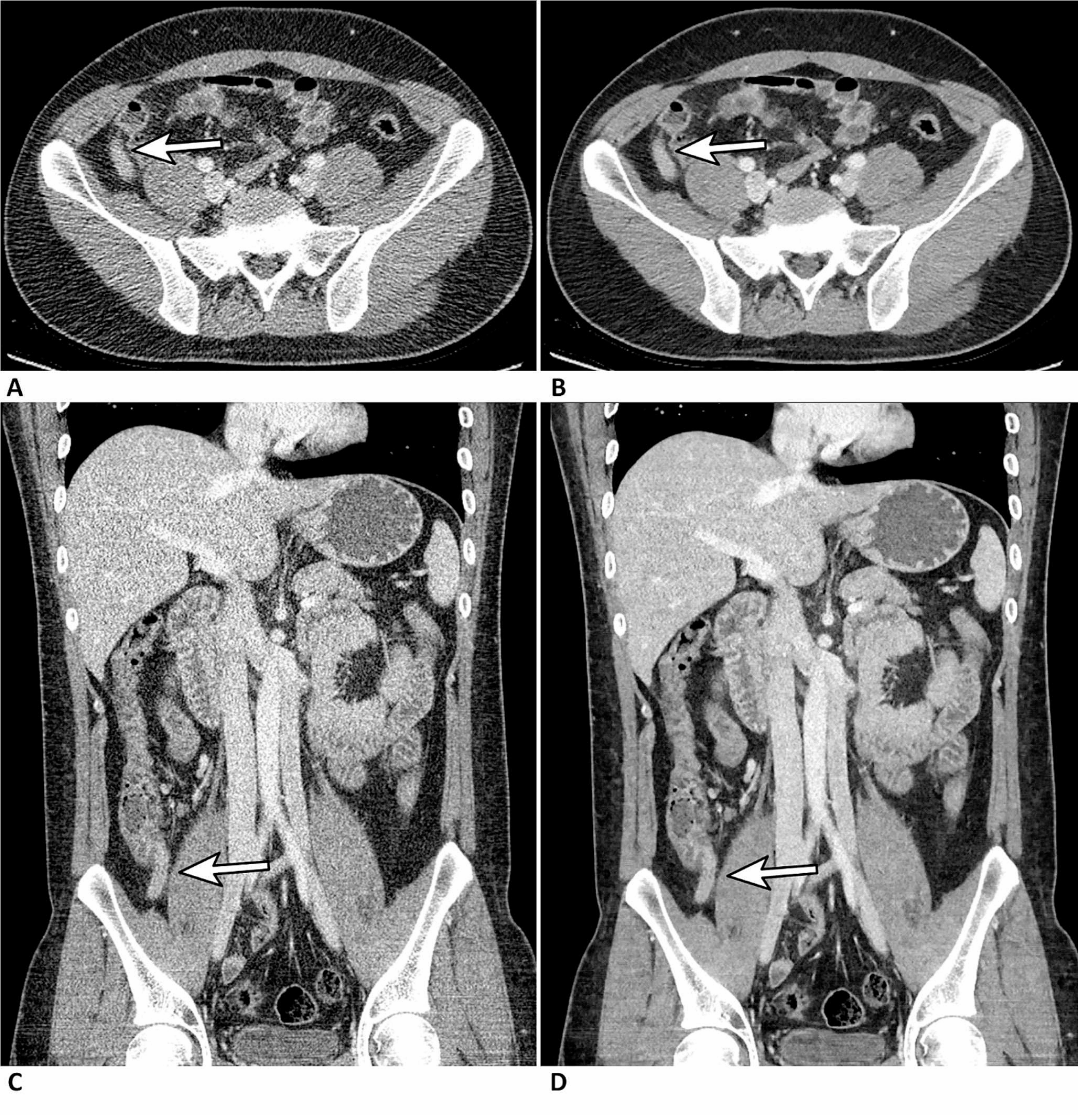
A 29-year-old man underwent CT examination for suspected appendicitis (64-channel CT from GE). Ultralow-dose CT images (**a, c**) and ultralow-dose CT with deep-learning denoising algorithm (**b, d**). The patient was confirmed with appendicitis through an appendectomy

The appendix was well visualized (appendiceal visualization score 2) in most patients (77.8–91.7% on ULDCT and 79.4–92.2% on D-ULDCT) (Fig. 2). Appendiceal visualization scores of all readers were not significantly different between ULDCT and D-ULDCT (Supplementary Table 3).

**Fig. 3:**
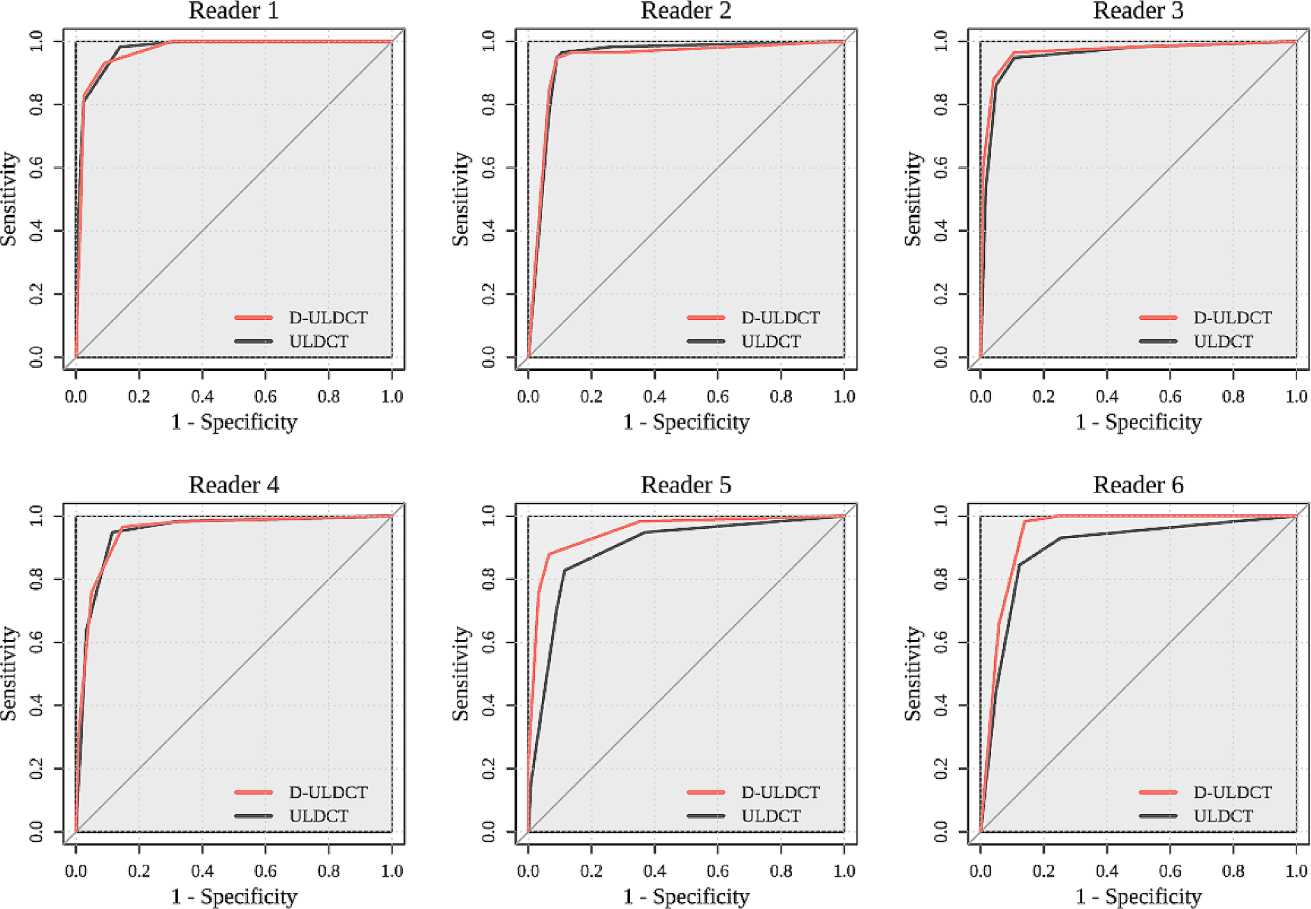
Receiver Operating Characteristic Curves for diagnosing acute appendicitis across readers (reader 1, 2, 3, board-certified radiologists; reader 4, third-year resident; reader 5, 6, second-year residents)

**Fig. 4:**
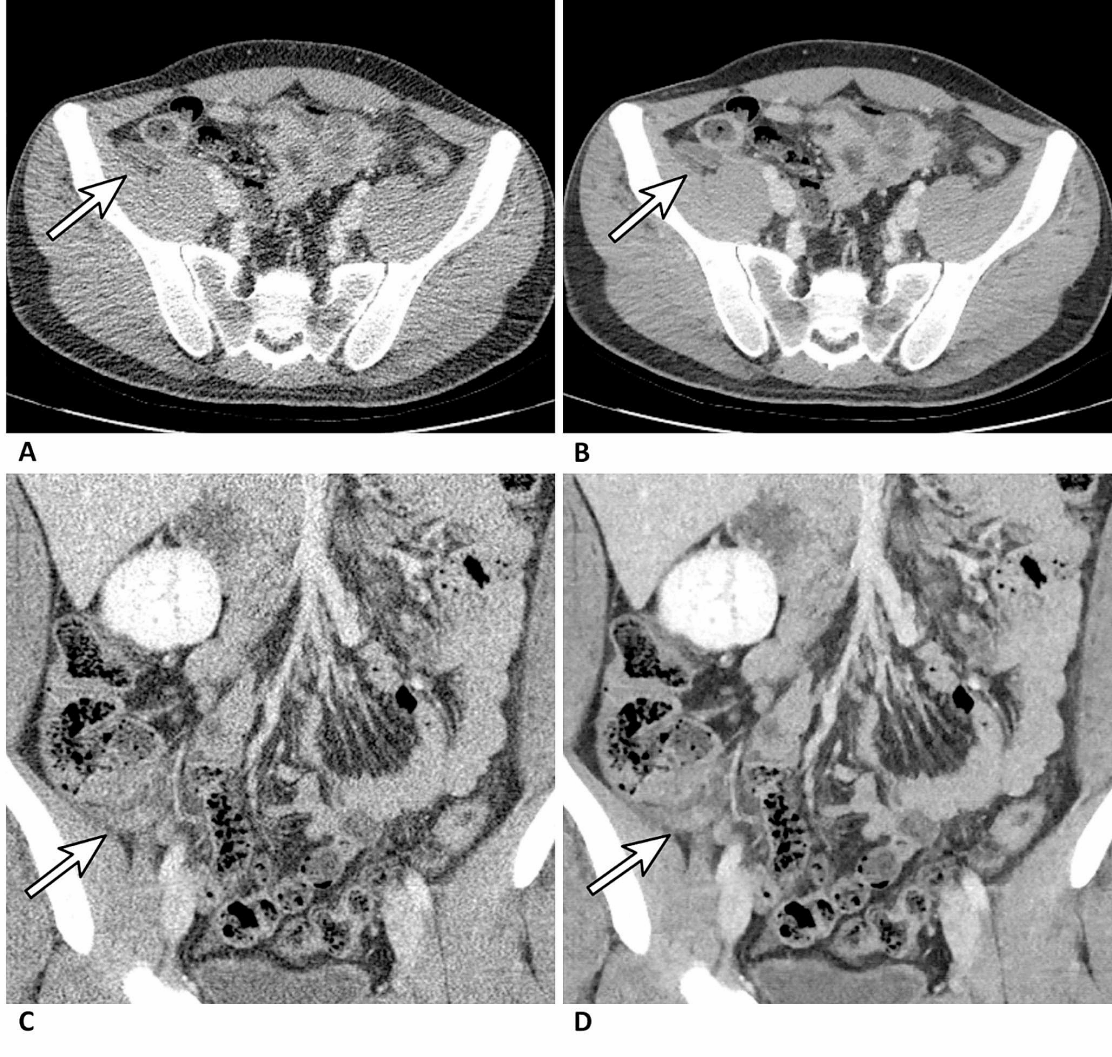
Representative images that an image-based deep-learning denoising algorithm improved diagnostic accuracy. A 37-year-old woman underwent a CT examination for suspected appendicitis (64-channel CT from Siemens). A second-year radiology resident (reader 6) diagnosed terminal ileitis on ultralow-dose CT images (**a, c**) and appendicitis on ultralow-dose CT with deep-learning denoising algorithm (**b, d**). The patient was confirmed with appendicitis through an appendectomy

The AUC of diagnosing appendicitis in readers ranged 0.90–0.97 and 0.94–0.97 on ULDCT and D-ULDCT, respectively (Fig. 3). The diagnostic performance of three board-certified radiologists (reader 1, reader 2, and reader 3) and one third-year resident (reader 4) for appendicitis did not differ significantly between ULDCT and D-ULDCT. However, for two second-year residents, diagnostic performance was significantly higher in D-ULDCT than in ULDCT (AUC difference of reader 5: 0.06 [95% CI, 0.01–0.11; *p* = .022]; AUC difference of reader 6: 0.05 [95% CI, 0.00–0.10; *p* = .046]) (Fig. 4; Table 2). On ULDCT, the diagnostic sensitivity of each reader ranged from 83 to 98%, and the specificity ranged from 75 to 89%. On D-ULDCT, the diagnostic sensitivity ranged from 88 to 100%, and the specificity ranged from 75 to 93% (Supplementary Table 4). The interobserver agreement for likelihood of appendicitis between ULDCT and D-ULDCT showed no significant difference in board-certified radiologists (0.87 vs. 0.86; 0.01 [95% CI, -0.05–0.07; *p* = .76]) and residents (0.68 vs. 0.72; -0.05 [95% CI, -0.14–0.05; *p* = .32]).

**Table 2:** Areas under the receiver operating characteristic curve for diagnosing acute appendicitis across readers

Reader	AUC		AUC difference (Confidence interval)	*P*-value
	ULDCT	D-ULDCT		
Reader 1	0.97	0.97	-0.00 (-0.02, 0.02)	0.68
Reader 2	0.95	0.94	-0.01 (-0.04, 0.03)	0.74
Reader 3	0.95	0.97	0.01 (-0.01, 0.03)	0.30
Reader 4	0.95	0.95	0.00 (-0.01, 0.02)	0.74
Reader 5	0.90	0.95	0.06 (0.01, 0.11)	0.022
Reader 6	0.90	0.95	0.05 (0.00, 0.10)	0.046

AUC difference = D-ULDCT - ULDCT. *AUC* Areas under the receiver operating characteristic curve, *ULDCT* Ultralow-dose CT, *D-ULDCT* Ultralow-dose CT with deep-learning denoising algorithm

All readers evaluated that D-ULDCT images using the image-based deep-learning denoising algorithm had lesser subjective image noise and better diagnostic acceptability (*p* < .001; except *p*-value of reader 3 on diagnostic acceptability = 0.003, Table 3). Regarding the artificial sensation, the evaluation from three board-certified radiologists indicated that D-ULDCT images were perceived as more artificial. Contrarily, the assessment from three radiology residents suggested that D-ULDCT images appeared more natural (*p* < .001).

**Table 3:** Qualitative image analysis

	Reader	ULDCT (median [IQR])	D-ULDCT (median [IQR])	*P*-value
Subjective image noise	Reader 1	2 [2, 2]	4 [4, 4]	< 0.001
	Reader 2	2 [2, 3]	3 [3, 4]	< 0.001
	Reader 3	2 [1, 2]	3 [2, 3]	< 0.001
	Reader 4	2 [2, 3]	3 [3, 3]	< 0.001
	Reader 5	2 [2, 3]	3 [3, 3]	< 0.001
	Reader 6	3 [2, 3]	3 [3, 3]	< 0.001
Diagnostic acceptability	Reader 1	2 [2, 2]	4 [4, 4]	< 0.001
	Reader 2	3 [3, 3]	3 [3, 3]	0.003
	Reader 3	2 [2, 2]	3 [2, 3]	< 0.001
	Reader 4	2 [2, 3]	3 [3, 3]	< 0.001
	Reader 5	3 [3, 3]	3 [3, 3]	< 0.001
	Reader 6	4 [3, 4]	4 [4, 4]	< 0.001
Artificial sensation	Reader 1	4 [4, 4]	3 [3, 3]	< 0.001
	Reader 2	4 [3, 4]	2 [2, 3]	< 0.001
	Reader 3	3 [3, 4]	3 [3, 4]	< 0.001
	Reader 4	4 [4, 5]	5 [5, 5]	< 0.001
	Reader 5	3 [3, 4]	4 [4, 4]	< 0.001
	Reader 6	3 [2, 3]	3 [3, 3]	< 0.001

*ULDCT* Ultralow-dose CT, *D-ULDCT* Ultralow-dose CT with deep-learning denoising algorithm, *IQR* interquartile range

### Quantitative image analysis

D-ULDCT images showed significantly lower image noise, higher SNR, and higher CNR (*p* < .001) compared to ULDCT images (Table 4). The image noise was lower on D-ULDCT than on ULDCT for the hepatic parenchyma (17.3 ± 6.5 vs. 42.0 ± 15.1, *p* < .001), paraspinal muscle (15.9 ± 6.0 vs. 37.4 ± 13.4, *p* < .001), and abdominal aorta (21.5 ± 8.0 vs. 50.0 ± 17.9, *p* < .001). The SNR was higher on D-ULDCT than on ULDCT for the hepatic parenchyma (8.6 ± 3.2 vs. 3.5 ± 1.3, *p* < .001), paraspinal muscle (4.9 ± 1.6 vs. 2.1 ± 0.7, *p* < .001), and abdominal aorta (10.1 ± 4.1 vs. 4.3 ± 1.7, *p* < .001). The CNR was higher on D-ULDCT than on ULDCT for the hepatic parenchyma (16.4 ± 5.8 vs. 8.1 ± 2.5, *p* < .001), paraspinal muscle (12.0 ± 4.1 vs. 5.9 ± 1.8, *p* < .001), and abdominal aorta (20.6 ± 7.5 vs. 10.1 ± 3.3, *p* < .001). The image noise seemed to increase and SNR/CNR seemed to decrease, according to body mass index (Supplementary Table 5).

**Table 4:** Quantitative image analysis

	Site	ULDCT (mean ± SD)	D-ULDCT (mean ± SD)	*P*-value
Image noise, HU	Liver	42.0 ± 15.1	17.3 ± 6.5	< 0.001
	Muscle	37.4 ± 13.4	15.9 ± 6.0	< 0.001
	Aorta	50.0 ± 17.9	21.5 ± 8.0	< 0.001
SNR (signal-to-noise ratio)	Liver	3.5 ± 1.3	8.6 ± 3.2	< 0.001
	Muscle	2.1 ± 0.7	4.9 ± 1.6	< 0.001
	Aorta	4.3 ± 1.7	10.1 ± 4.1	< 0.001
CNR (contrast-to-noise ratio)	Liver	8.1 ± 2.5	16.4 ± 5.8	< 0.001
	Muscle	5.9 ± 1.8	12.0 ± 4.1	< 0.001
	Aorta	10.1 ± 3.3	20.6 ± 7.5	< 0.001

*ULDCT* Ultralow-dose CT, *D-ULDCT* Ultralow-dose CT with deep-learning denoising algorithm, *SD* standard deviation

## Discussion

In this study, an image-based deep-learning denoising algorithm improved image quality of ultralow-dose CT in diagnosing acute appendicitis and the diagnostic performance of less-experienced radiology residents.

Our study demonstrates several strengths in managing dose reduction in CT, which is a challenging situation in clinical practice. First, our analysis applies the image-based deep-learning algorithm to reconstructed CT images, not sinograms, ensuring generalizability. Previous studies have reported improvements in qualitative or quantitative features on CT images with deep learning-based reconstruction algorithms compared to filtered back projection or iterative reconstruction techniques [9, 29–32]. However, most of these studies relied on sinograms for applying deep learning-based reconstruction. In contrast, our study used image-based denoising algorithm, providing a practical approach to dose reduction even in cases where sinograms cannot be obtained. We attributed this capability to the denoising algorithm trained on a large dataset of image data. Previous studies demonstrated its ability to improve image quality and diagnostic accuracy in low-dose coronary CT angiography [17], show the best overall image quality with fewer artifacts in ultralow-dose chest CT when compared with vendor-specific deep learning image reconstruction [23], and maintain image quality while reducing radiation dose and iodine administration in pediatric abdominal CT examinations [24]. Second, we analyzed image data from various CT machines from different vendors. Previous feasibility study with 30 patients had reported that ULDCT would be non-inferior to 2-mSv CT for diagnosing appendicitis [33]. However, the study had limitations, including a small sample size with only one CT machine. On the other hand, our results demonstrated promising applicability, versatility, and the potential for broad implementation across larger patients and various CT machines. Third, we revealed the usefulness of the image-based deep learning denoising algorithm in the clinical task of diagnosing appendicitis. This aspect goes beyond a simple image quality comparison between ULDCT and D-ULDCT. There might be a certain gap between improving image quality and assisting diagnosis. Thus, our findings showed that the practical application of the deep-learning denoising algorithm could help enhance diagnostic accuracy, especially for less-experienced radiologists.

Interestingly, in terms of appendiceal visualization, we did not observe a significant difference between ULDCT and D-ULDCT. The appendix was well visualized in most patients on both the ULDCT and D-ULDCT images, with a range of 77.8–91.7% for ULDCT and 79.4–92.2% for D-ULDCT, respectively. Considering that a previous study achieved good visualization in more than 85% of cases with 2-mSv CT [34], appendiceal visualization might be slightly compromised, although the formal statistical comparison is limited. However, regardless of the degree of appendix visualization, radiologists achieved a good diagnostic performance (AUC, 0.94–0.97) for appendicitis in D-ULDCT, which aligns with the previous study’s findings [34]. Intriguingly, radiologists could accurately diagnose or rule out appendicitis in most cases, even if the appendix was not visualized. While we cannot provide a definitive explanation for this finding, we assume that radiologists possibly relied on secondary findings, such as fat infiltration or fluid collection in the right lower quadrant of the abdomen, to aid in their diagnostic decision-making.

Regarding the artificial sensation in denoised images, board-certified abdominal radiologists perceived D-ULDCT as more artificial, whereas radiology residents found it more natural. We hypothesized that the images from D-ULDCT might be less familiar and more artificial to experienced radiologists who are accustomed to reading CT images with filtered back projection or iterative reconstruction. On the other hand, D-ULDCT might seem more clear and natural to less experienced residents compared to ULDCT, and this partially contributed to the finding that D-ULDCT improved the diagnostic accuracies of the two residents.

Our study had limitations. First, ultralow-dose CT images were simulated from the 2-mSv CT images using a validated noise insertion tool [15]. While this allowed us to compare images from the same patients at different radiation doses without additional exposure, it is important to note that the simulated images may not perfectly replicate the original data. Thus, the results obtained from the simulated images may not completely reflect the outcomes observed in actual CT examinations. Second, although we drew the ROI for various organs for quantitative analysis, there may be potential bias in measuring the mean Hounsfield unit, particularly for small objects. Third, our study focused solely on evaluating the deep learning denoising algorithm and did not include a comparison with other CT reconstruction methods, such as filtered back projection or iterative reconstruction. Future studies could benefit from including such comparisons to provide a more comprehensive understanding of the performance and effectiveness of different reconstruction techniques.

In conclusion, an image-based deep-learning denoising algorithm can provide better image quality of ultralow-dose CT in diagnosing acute appendicitis and enhance diagnostic performance for less-experienced radiologists.

### Electronic supplementary material

Below is the link to the electronic supplementary material.

Supplementary Material 1
