# Supplementary material for: Diagnostic performance and image quality of an image-based denoising algorithm applied to radiation dose-reduced CT in diagnosing acute appendicitis
Source: Abdom Radiol (NY). 2024 Feb 27;49(6):1839–49. doi: 10.1007/s00261-024-04246-3 (PMC11213764; doi:10.1007/s00261-024-04246-3)
Supplement: Supplementary file 1 — Supplementary Material 1 [file 261_2024_4246_MOESM1_ESM.docx]

**Supplementary materials**

| **Supplementary Table 1** Standardized CT report form of appendiceal visualization and likelihood score for appendicitis | |
| --- | --- |
| Analyzed findings and scoring criteria | |
| Visualization of the appendix | |
|  | Grade 0. Not identified. |
|  | Grade 1. Unsure or partly visualized. |
|  | Grade 2. Clearly and entirely visualized. |
| Likelihood of appendicitis | |
|  | Grade 1. Definitely absent. Clinical observation is recommended. |
|  | Grade 2. Probably absent. Clinical observation is recommended. |
|  | Grade 3. Indeterminate. Clinical observation or surgical exploration is recommended. |
|  | Grade 4. Probably present. Surgical exploration is recommended. |
|  | Grade 5. Definitely present. Surgical exploration is recommended. |
| Appendiceal perforation | |
|  | Grade 0. Unlikely present |
|  | Grade 1. Equivocal |
|  | Grade 2. Likely present |
| Periappendiceal abscess that needs a drainage procedure | |
|  | Absent or present |
| Alternative diagnosis | |

| **Supplementary Table 2** Scoring system for various parameters in the qualitative image analysis of each CT image | | | |
| --- | --- | --- | --- |
| Image quality parameter |  | Score | Scoring system |
| Subjective image noise | Degree of mottling or graininess in the images | 1–5 | Score 1, “unacceptable noise”; |
|  |  |  | Score 2, “above-average increased noise”; |
|  |  |  | Score 3, “average noise”; |
|  |  |  | Score 4, “less-than-average noise”; |
|  |  |  | Score 5, “minimal or no image noise” |
| Diagnostic acceptability | Reader’s confidence in making a reasonable diagnosis from the images | 1–5 | Score 1, “diagnostically unacceptable”; |
|  |  |  | Score 2, “subdiagnostic for a diagnosis”; |
|  |  |  | Score 3, “average”; |
|  |  |  | Score 4, “better than average”; |
|  |  |  | Score 5, “excellent” |
| Artificial sensation | Degree of plastic-looking, smooth, paint-brushed, or unnatural texture | 1–5 | Score 1, “a severe degree of plastic-looking, smooth, paint-brushed, or unnatural texture”; |
|  |  |  | Score 5, “a minimal degree of plastic-looking, smooth, paint-brushed, or unnatural texture” |

| **Supplementary Table 3** Appendiceal visualization score of readers | | | |  |
| --- | --- | --- | --- | --- |
|  | Reader | ULDCT  (median [IQR]) | D-ULDCT  (median [IQR]) | P-value |
| Appendiceal visualization | Reader 1 | 2 [2, 2] | 2 [2, 2] | .23 |
|  | Reader 2 | 2 [2, 2] | 2 [2, 2] | .84 |
|  | Reader 3 | 2 [2, 2] | 2 [2, 2] | .58 |
|  | Reader 4 | 2 [2, 2] | 2 [2, 2] | .60 |
|  | Reader 5 | 2 [2, 2] | 2 [2, 2] | .40 |
|  | Reader 6 | 2 [2, 2] | 2 [2, 2] | > .99 |
| *ULDCT* Ultralow-dose CT, *D-ULDCT* Ultralow-dose CT with deep-learning denoising algorithm, *IQR* interquartile range. | | | | |

| **Supplementary Table 4** Diagnostic sensitivity and specificity of readers for appendicitis | | | | | |
| --- | --- | --- | --- | --- | --- |
| Reader | Sensitivity | |  | Specificity | |
|  | ULDCT | D-ULDCT |  | ULDCT | D-ULDCT |
| Reader 1 | 98% (57/58) | 93% (54/58) |  | 86% (105/122) | 91% (111/122) |
| Reader 2 | 97% (56/58) | 97% (56/58) |  | 89% (109/122) | 86% (105/122) |
| Reader 3 | 95% (55/58) | 97% (56/58) |  | 89% (109/122) | 89% (109/122) |
| Reader 4 | 95% (55/58) | 97% (56/58) |  | 89% (108/122) | 85% (104/122) |
| Reader 5 | 83% (48/58) | 88% (51/58) |  | 89% (108/122) | 93% (114/122) |
| Reader 6 | 93% (54/58) | 100% (58/58) |  | 75% (91/122) | 75% (92/122) |
| *ULDCT* Ultralow-dose CT, *D-ULDCT* Ultralow-dose CT with deep-learning denoising algorithm. | | | | | |

| **Supplementary Table 5** Quantitative image analysis according to body mass index | | | | | | | |
| --- | --- | --- | --- | --- | --- | --- | --- |
| Body mass index, kg/m^2^ | | <18.5 (underweight) | | 18.5–24.9 (normal) | | ≥25.0 (overweight) | |
|  | Site | ULDCT | D-ULDCT | ULDCT | D-ULDCT | ULDCT | D-ULDCT |
| Image noise, HU | Liver | 32.4 ± 7.5 | 13.4 ± 3.1 | 40.7 ± 13.9 | 16.7 ± 5.9 | 49.7 ± 18.5 | 20.7 ± 8.2 |
|  | Muscle | 29.5 ± 6.6 | 12.4 ± 2.6 | 36.6 ± 11.8 | 15.4 ± 5.0 | 42.8 ± 18.5 | 18.6 ± 8.7 |
|  | Aorta | 41.3 ± 8.4 | 17.5 ± 3.8 | 48.8 ± 17.1 | 20.9 ± 7.6 | 57.2 ± 21.1 | 25.0 ± 9.3 |
| SNR (signal-to-noise ratio) | Liver | 4.7 ± 0.8 | 11.3 ± 2.2 | 3.7 ± 1.2 | 9.0 ± 2.9 | 2.5 ± 1.2 | 6.1 ± 3.0 |
|  | Muscle | 2.5 ± 0.6 | 5.9 ± 1.4 | 2.1 ± 0.7 | 5.0 ± 1.5 | 1.9 ± 0.7 | 4.4 ± 1.7 |
|  | Aorta | 4.9 ± 0.8 | 11.7 ± 2.2 | 4.4 ± 1.7 | 10.5 ± 4.0 | 3.5 ± 1.9 | 8.0 ± 4.3 |
| CNR (contrast-to-noise ratio) | Liver | 9.7 ± 2.7 | 17.6 ± 6.5 | 8.3 ± 2.5 | 16.9 ± 5.7 | 6.6 ± 2.2 | 14.2 ± 5.4 |
|  | Muscle | 6.6 ± 2.4 | 11.7 ± 4.6 | 6.0 ± 1.8 | 12.2 ± 4.1 | 5.4 ± 1.7 | 11.5 ± 4.1 |
|  | Aorta | 11.9 ± 3.8 | 21.3 ± 6.9 | 10.4 ± 3.2 | 21.1 ± 7.7 | 8.5 ± 3.0 | 18.1 ± 6.8 |
| Data are presented as mean ± standard deviation. *ULDCT* Ultralow-dose CT, *D-ULDCT* Ultralow-dose CT with deep-learning denoising algorithm. | | | | | | | |
